# Fluidization of nanopowders: a review

**DOI:** 10.1007/s11051-012-0737-4

**Published:** 2012-02-10

**Authors:** J. Ruud van Ommen, Jose Manuel Valverde, Robert Pfeffer

**Affiliations:** 1 Department of Chemical Engineering, Delft University of Technology, Julianalaan 136, 2628 BL Delft, The Netherlands; 2 Department of Electronics and Electromagnetism, University of Seville, Avenida Reina Mercedes s/n, 41012 Sevilla, Spain; 3 Chemical Engineering Program, School for Engineering of Matter, Transport, and Energy, Arizona State University, Tempe, AZ 85287 USA

**Keywords:** Nanoparticles, Agglomerates, Fluidized beds, Assisted fluidization, Modeling nanofluidization, Literature review

## Abstract

Nanoparticles (NPs) are applied in a wide range of processes, and their use continues to increase. Fluidization is one of the best techniques available to disperse and process NPs. NPs cannot be fluidized individually; they fluidize as very porous agglomerates. The objective of this article is to review the developments in nanopowder fluidization. Often, it is needed to apply an assistance method, such as vibration or microjets, to obtain proper fluidization. These methods can greatly improve the fluidization characteristics, strongly increase the bed expansion, and lead to a better mixing of the bed material. Several approaches have been applied to model the behavior of fluidized nanopowders. The average size of fluidized NP agglomerates can be estimated using a force balance or by a modified Richardson and Zaki equation. Some first attempts have been made to apply computational fluid dynamics. Fluidization can also be used to provide individual NPs with a thin coating of another material and to mix two different species of nanopowder. The application of nanopowder fluidization in practice is still limited, but a wide range of potential applications is foreseen.

**Electronic supplementary material:**

The online version of this article (doi:10.1007/s11051-012-0737-4) contains supplementary material, which is available to authorized users.

## Introduction

Nanoscience has attracted much attention from researchers over the past decades, but true nanotechnology has only more recently begun to bestow promising results for a wide range of applications. It has brought advances such as energy-efficient LED lighting (Krames et al. 2007) and improved catalysts (Bell 2003; Li and Somorjai 2010), and is beginning to deliver medical breakthroughs (Riehemann et al. 2009). Nanotechnology encompasses the study and application of objects with at least one dimension smaller than 100 nm. Nanoparticles (NPs)—with all three dimensions below 100 nm—have been widely studied over the past two decades, since their large surface area per unit mass leads to unique chemical, electro-magnetic, optical, and other properties.

For many practical applications of NPs, it is required to have large amounts of the material. Many of the synthesis and processing techniques for NPs that are currently under research—most of them operating in the liquid phase—are just aimed at small quantities. We think that it is important to consider the potential for scaling up right from the start; this is typically easier in the gas phase than in the liquid phase. Gas phase methods offer inherent advantages such as the absence of solvent waste, less separation problems, the feasibility of continuous processing as opposed to batch processing, and the versatility with respect to particle material and size and structure (Kruis et al. 1998; El-Shall and Schmidt-Ott 2006).

For the processing of micron-sized particles, a widely applied technique is fluidization: suspending the particles in an upward gas stream with such a velocity that drag and gravity are in equilibrium. Although it may sound counterintuitive, nanopowders can be fluidized as well. In contrast to particles of say 200 μm, however, NPs are not fluidized individually but as agglomerates: very dilute clusters of around 200 μm consisting of ~10^10^ primary particles. The fluidization of nanopowders has attained increasing attention in the past decade. The objective of this article is to review the developments in the field.

## The agglomerating nature of NPs in the gas phase

### Forces between NPs

**Fig. 1:**
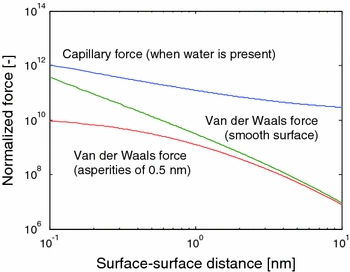
The main forces between two silica particles of 10 nm as a function of the interparticle distance. All forces are normalized by dividing them by the gravity forces on a single particle. The capillary force is given for water; for other liquids, this force is typically lower. The van der Waals force depends on the surface roughness, as shown by the curves for a smooth surface and for surface asperities. Models and constants from Butt and Kappl (2010)

The three main interactions between particles in the gas phase are van der Waals interaction, liquid bridging, and electrostatic interaction (Seville et al. 2000). Capillary or liquid bridges can be formed due to liquid that is adsorbed on the particle surface. When these bridges are formed, they normally dominate the interaction (see Fig. 1), but this is strongly dependant on the presence of liquid and the contact angle. The influence of capillary bridging on NP fluidization has not yet been studied in detail; in most cases, the van der Waals forces are assumed to be most important. The electrostatic charge strongly depends on previous interaction with other materials (tribocharging) and is typically less relevant at this small scale. It can, however, play an important role as a force between agglomerates.

In the liquid phase, several mechanisms can overpower the van der Waals forces and prevent clustering of NPs, e.g., double layers formed by an electrolyte and steric hindrance by dissolved polymers. In the gas phase, separation mechanisms are less widespread, and the Hamaker constant—determining the magnitude of the van der Waals force—is in general larger than in the liquid phase (Butt and Kappl 2010). Therefore, NPs in the gas phase will typically have the tendency to agglomerate, unless they are charged. The nature of the particle surfaces will influence the van der Waals forces between the particles in different ways. First, the presence of a different material will lead to a different Hamaker constant. Second, the surface roughness might be changed, which also influences the van der Waals forces, as illustrated in Fig. 1. The van der Waals force between particles (diameter *d*_p_) with asperities of size *r*_asp_ is given by Castellanos (2005):

1 $$ F_{\text{vdW}} = \frac{{A_{\text{H}} d_{\text{p}}^{3} }}{{12(x + r_{\text{asp}} )^{2} (x + r_{\text{asp}} + d_{\text{p}} )^{2} }} $$

where *A*_H_ is the Hamaker constant and *x* is the surface–surface distance. This equation is often simplified as:

2 $$ F_{\text{vdW}} = \frac{{A_{\text{H}} d_{\text{p}} }}{{12\;x^{2} }} $$

The formed agglomerates—in which the particle–particle bonds are not permanent—should be distinguished from aggregates, in which the particles are bound more strongly by solid-state necks (Teleki et al. 2008b). However, in many production processes, such as the widely used flame synthesis, high temperatures are involved that lead to indestructible aggregates of NPs by fusing of the contacts (Seipenbusch et al. 2010); these aggregates are typically of the order of one micron or smaller. Some authors use the term soft agglomerates versus hard agglomerates instead of agglomerates versus aggregates (Nichols et al. 2002), while others use the terms interchangeably. The agglomerating nature of NPs in the gas phase is not just detrimental: it actually makes it possible to process large amounts of nanoparticulate material in small volumes.

### The fractal morphology of NP agglomerates

The nature of NP agglomerates has been widely studied outside the fluidization literature. With the introduction of the concept of fractal geometry by Mandelbrot (1982), a proper way evolved to describe these agglomerates (Bushell et al. 2002). A fractal object shows self-similarity under transformation of scale (e.g., changing the magnification of a microscope). The number of particles in an agglomerate *N* scales as (Friedlander 2000):

3 $$ N\sim \left( {\frac{{r_{\text{aggl}} }}{{r_{\text{part}} }}} \right)^{D} $$

where *r*_aggl_ is the agglomerate radius, *r*_part_ is the particle radius, and *D* is the fractal dimension. For a compact agglomerate *D* approaches 3, but NP agglomerates are typically more dilute with a fractal dimension *D* < 3. Forrest and Witten Jr. (1979) were the first to report the fractal nature of NP agglomerates. Later, it was shown that the detailed chemical nature of the NPs has little influence on the resulting agglomerates, but that the formation process does have a large effect (Lin et al. 1989; Schaefer 1989). Two general classes of agglomeration were distinguished, both starting from single particles: particle–cluster agglomeration and cluster–cluster agglomeration. Note that most authors describing these mechanisms use the term aggregation rather than agglomeration.

**Fig. 2:**
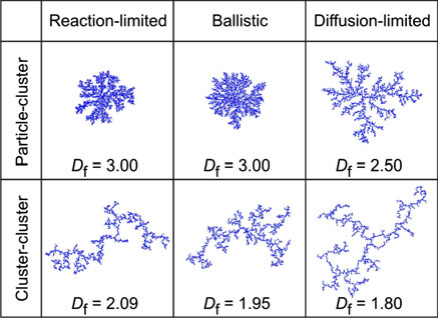
Kinetic growth models in a 2D embedding space. The mass fractal dimension D of their 3D analogs are given (based on Friedlander (2000))

In the case of particle–cluster agglomeration, the clusters, once formed, no longer move and all agglomeration is due to accretion of single particles. In the case of cluster–cluster agglomeration, the clusters themselves continue to move, collide, and form yet larger clusters. This yields a very complex distribution of clusters of different sizes. Within each class, three different regimes can be distinguished: reaction-limited agglomeration (RLA), diffusion-limited agglomeration (DLA), and ballistic agglomeration (BA). In case of RLA, there is some form of repulsive interaction between approaching particles, so that only a small portion of the collision leads to agglomeration. In case of DLA or BA, every collision results in particles or clusters sticking together. In DLA, the particles (or clusters) experience Brownian motion, whereas in BA they follow linear trajectories. Each class and regime leads to a specific morphology and fractal dimension of the agglomerate, as shown in Fig. 2.

**Fig. 3:**
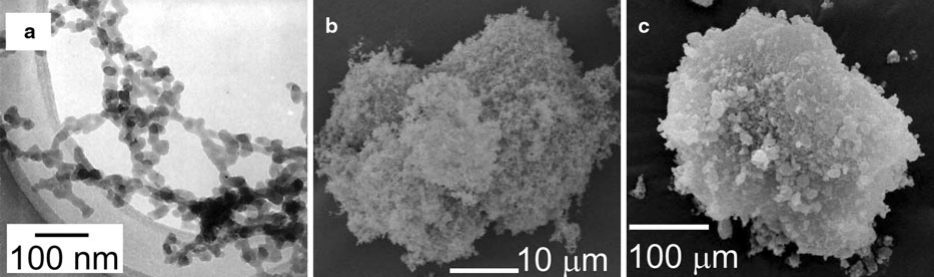
Illustration of the multistage agglomerate structure obtained by ex-situ analysis. **a** TEM image of a network of silica NPs. **b** SEM image of a simple agglomerate or sub-agglomerate built up from these networks. **c** SEM image of a complex agglomerate consisting of several sub-agglomerates (reprinted from Wang et al. (2002) with permission from Elsevier)

Nam et al. (2004) were the first to experimentally estimate the fractal dimension of fluidized NP agglomerates, based on earlier work on fine powders by Valverde et al. (2001b). They found fractal dimensions around 2.57, close to the value of 2.5 that was earlier found from simulations for particle–cluster DLA. Also, the structure found from TEM analysis by Wang et al. (2002) (see Fig. 3a) shows the best agreement with the simulated structure for particle–cluster DLA. It is, however, remarkable that this is the prevailing mechanism and not cluster–cluster explanation. It might be due to the fact that “simple agglomerates” (small agglomerates, see below) are already formed before fluidization, combining to larger agglomerates during fluidization.

Wang et al. (2002) analyzed more in detail the fluidization and agglomerate structure of six kinds of silica powders, with primary particles size from 7 to 16 nm. By applying the Richardson–Zaki (R–Z) equation to bed expansion measurement, they determined the average agglomerate size to be 230–330 μm; the void fraction is as high as 98–99%. Wang et al. (2006b) argued that this direct application of the R–Z equation may lead to an overestimation of the mean terminal velocity of the agglomerates, and thus in an overestimation of the size and/or an underestimation of the agglomerate voidage. Wang et al. (2002) also reported that the agglomerates have a multistage structure. They show using TEM that on the smallest scale silica NPs form 3D netlike structures (Fig. 3a). These netlike structures, with sizes around 1 μm, may be hold together by van der Waals forces, but the particles can also be connected by solid inter-particle necks, depending on the method used to synthesize the particles. The netlike structures coalesce into larger conglomerations, with the shape of a single sphere or ellipsoid, which they call “simple agglomerate.” These simple agglomerates typically have sizes of 1–100 micron (see Fig. 3b). However, this size range is too small in comparison with agglomerate diameters determined from fluidization experiments.

Nam et al. (2004) aspirated samples of silica nanoagglomerates at different heights out of their expanded fluidized bed and examined them under the SEM. The agglomerate sizes averaged only around 30 μm, and the agglomerates were very porous and fragile. It appeared that the larger fluidized agglomerates probably were broken down into smaller simple agglomerates during their removal from the bed and/or during sample preparation for the SEM.

Wang et al. (2002) concluded that simple agglomerates should form complex agglomerates during fluidization, with sizes ranging from 200–400 μm. They also show such agglomerates using TEM (see Fig. 3c), but it is uncertain whether these agglomerates exactly look like the ones inside the fluidized bed. Wang et al. (2002) did not speculate whether only the netlike structure has a fractal nature or that this is also found at larger scales. Wang et al. (2006b) put forward three critical remarks about the correctness of the results of Wang et al. (2002). First, the samples could be increasingly consolidated if they were left inside the bed for too long. Second, in the process of getting the samples out of the bed for electron microscopy, the samples could be contaminated by particles resting near the sampling ports. Third, for the imaging, the sample had to undergo treatments, which could alter the original structure. As an alternative, Wang et al. (2006b) proposed laser-based planar imaging of agglomerates just above the bed surface. This will be discussed in the section “Determination of the agglomerate size”.

## Fluidization of nanopowders using aeration alone

Many nanopowders form large and compact agglomerates simply due to storage and are very difficult to fluidize because of the large cohesive forces between the particles, given their size and extensive surface area. Therefore, removing agglomerates larger than 500 μm will usually improve fluidization quality. Some nanopowders will fluidize smoothly at low superficial velocities with practically no bubbles, large bed expansion, and little elutriation. Other nanopowders require relatively high superficial velocities to be fluidized, and vigorous bubbling with significant elutriation is observed. To smoothly fluidize and process these types of nanopowders without considerable gas-bypassing, some sort of external assistance such as vibration or stirring is usually required. We will treat the various assistance methods later in this article; in this section, we will discuss gas fluidization of nanopowders without assistance methods.

Chaouki et al. (1985) were one of the first to report the fluidization of aerogel (highly porous aggregates of primary particles a few nanometers in size). They showed that nanostructured Cu/Al_2_O_3_ aerogel fine particles can be smoothly fluidized at superficial velocities greatly in excess of the expected minimum fluidization velocity for such fine powders, because they form stable clusters or agglomerates. These agglomerates fluidized uniformly and expanded in a homogeneous manner, providing a means of dispersing and processing the very high specific surface area nanostructured aerogels. Morooka et al. (1988) were able to fluidize submicron (20–500 nm) Ni, Si_3_N_4_, SiC, Al_2_O_3_, and TiO_2_ particles at high gas velocities. The particles formed agglomerates and large gas bubbles were observed. Similarly, Pacek and Nienow (1990) were also able to fluidize ultrafine, very dense, hard metal powders (particle diameter 2–8 μm), which formed agglomerates. At higher gas velocities, the bed had two layers: a bottom layer with large agglomerates (up to 2 mm in diameter) and a top layer of smaller agglomerates, which fluidized smoothly. At even higher gas velocities, the entire bed was fluidized and the large agglomerates were broken up into smaller, more stable ones. They also reported that the bed behaved as if fluidizing Geldart group B powders—bubbling occurred at the minimum fluidization velocity (*U*_mf_), and bed expansion was low. Song et al. (2009) showed that adding coarser particles (e.g., FCC catalyst) to a fluidized bed of NPs improves the fluidization quality: it increased the bed expansion and reduced the elutriation.

Wang et al. (2002) studied the fluidization of various fumed silica NPs. They showed that hydrophobic NPs expanded several times, from 2.5 up to 10 times their initial bed height and that hydrophilic NPs expanded only 1.5 up to 3 times their initial bed height. They also found relatively large minimum fluidization velocities for the hydrophilic NPs as compared to the hydrophobic particles. Wang et al. (2002) introduced the classification of the fluidization of nanopowders into “agglomerate particulate fluidization” (APF) and “agglomerate bubbling fluidization” (ABF); see Table 1 and the movies in the supplementary material. APF refers to smooth, liquid-like, bubble-less fluidization as previously observed when fluidizing aerogels (Chaouki et al. 1985). ABF refers to bubbling fluidization with very little bed expansion as previously observed by other researchers (Morooka et al. 1988; Pacek and Nienow 1990). ABF is observed not only for NPs, but also for other small particles of Geldart type C. APF is exclusively found for certain types of NPs and conditioned fine powders such as xerographic toners (Valverde and Castellanos 2007b). Wang et al. (2000) proposed to classify NPs exhibiting APF as E-particles, but this naming has never been adopted by other researchers.

**Table 1:** Comparison of the fluidization behavior of APF and ABF (based on Wang et al. 2002)

	APF	ABF
Primary particle size	Nanoparticles	Micro-, Submicro-, Nanoparticles
Bulk density	Low (<100 kg/m^3^)	High (>100 kg/m^3^)
Fluidization characteristics	1. Bubbleless	1. With bubbles
	2. With high bed expansion ratio	2. With low bed expansion ratio
	3. Agglomerates uniformly distributed in the bed	3. Large agglomerates at the bottoms of the bed, with small ones on the top
	4. Fluidized bed homogeneously expands, and the bed density decreases with increasing *U*_g_	4. Bed expansion ratio and emulsion phase density do not change much with increasing *U*_*g*_
Graphic representation	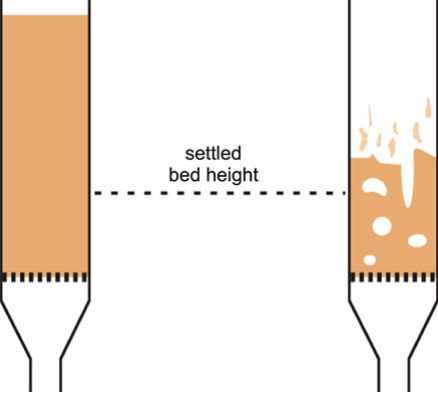	

Esmaeili et al. (2008) studied the solids hold-up distribution of zirconia and alumina particles of 250 and 120 nm diameters, respectively. They reported ABF-type behavior and found using optical fibers and radioactive densitometry that both in radial and axial direction, the solids hold-up is quite constant. Only for alumina, a change in the axial direction was found: the solids hold-up increased when moving in the upward direction. Esmaeili et al. (2008) suggest that this is due to larger agglomerates leading to larger bubbles in the bottom zone. However, this does not seem logical given the fact that larger bubbles will rise faster and lead to a higher gas solids hold-up. Further research will be required to elucidate this topic.

Wang et al. (2007b) state that NP fluidization does fit in the classical Geldart fluidization regime map, with A, B, C, and D powders (Geldart 1973). They report that agglomerates with typical properties (diameter of 220 μm and apparent density of 22 kg/m^3^) are close to the A/C boundary in the Geldart diagram: the ratio of the inter-agglomerate force to the buoyant weight of a single agglomerate is comparable to the same ratio for macro-sized particles at the AC boundary. This indicates why NPs sometimes show C-type behavior and other times show more A-type behavior (homogeneous fluidization).

**Fig. 4:**
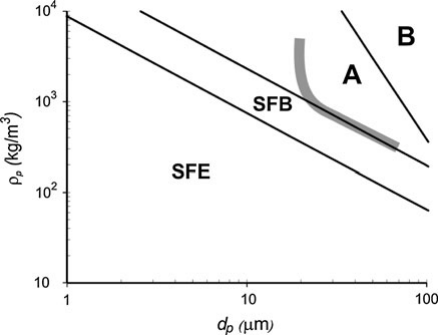
Modified Geldart’s diagram (Valverde and Castellanos 2007b) showing the boundaries between the types of fluidization expected for fine particles, including solid-to-fluid like to elutriation (*SFE*) behavior and solid-to-fluid like to bubbling (*SFB*) behavior. The thick gray line represents the boundary between A and C powders as shown in the original Geldart’s diagram (Geldart 1973)

Valverde and Castellanos (2007b) used a different approach: they utilized the similarity between the fluidization behavior of beds of non-cohesive particles fluidized by liquids and the uniform behavior of gas-fluidized beds of conditioned fine powders (Valverde et al. 2003; Wang et al. 2002). They used empirical relationships for liquid-fluidization of larger particles and modified them to take into account the agglomeration in gas fluidization of cohesive particles (see also the section “Modeling of NP fluidized beds”). They distinguished two different states of homogeneous fluidization: solid-like fluidization in which the agglomerates are jammed and keeping their place (mostly similar to homogeneous fluidization of Geldart A particles) and liquid-like fluidization in which agglomerates freely move, but no macroscopic bubbles are formed. With increasing gas velocity, NPs are moving from the solid-like to the fluid-like fluidization state. With a further increase of the gas velocity, very light and small NP agglomerates will be elutriated, while in the case of larger and heavier NPs (roughly *d*_p_ > 30 nm and *ρ*_p_ > 3,000 kg/m^3^), they will move from fluid-like to bubbling fluidization. This corresponds to APF and ABF behaviors, respectively. Using this approach, they defined solid-like to fluid-like to elutriation (SFE) behavior and solid-like to fluid-like to bubbling (SFB) behavior. These two types of behavior would replace the classical Geldart type C behavior for the new type of fluidizable fine and ultrafine powders, which were unknown at the time the classical Geldart diagram was reported (see Fig. 4).

### Determination of the agglomerate size

The formation of porous and light agglomerates is the key reason why NPs can be fluidized. To determine their fluidization characteristics, it is important to know the size of the agglomerates. Zhu et al. (2005) fluidized many different Evonik-Degussa Aerosil^®^ and Aeroxide^®^ metal oxide nanopowders (hydrophilic and hydrophobic silicas, alumina, and titania) as well as carbon blacks from Cabot Corp. conventionally (aeration alone). Some of these powders showed APF behavior, while others showed ABF-type behavior. They took images of the fluidized agglomerates at the interface between the bed and the freeboard (in the splash zone) with a CCD camera illuminated by a laser beam and used image analysis software to find the average agglomerate size. Zhu et al. (2005) also estimated the average agglomerate size from initial and final bed height measurements combined with the R–Z equation and obtained reasonably good agreement with the measured agglomerate sizes in the splash zone for APF-type nanopowders. For example, for Aerosil R974 (a hydrophobic silica showing APF behavior), the experimentally measured value of the agglomerate size was 315 μm as compared to 211 μm using the R–Z equation with *n* = 5.0.

Wang et al. (2006a) measured the size of fluidized agglomerates of Evonik-Degussa fumed silica Aerosil R974 in the splash zone by using a high-resolution CCD camera and a planar laser sheet for illumination. Their experimental equipment and image analysis algorithm provided more accurate images of the fluidized nanoagglomerates than previous studies. They reported both a number or length-based average (N-L) and a volume-based average (S-V) agglomerate size. Both the measured N-L and the S-V average agglomerate size varied with gas velocity, with an S-V average size of 262 μm at 1.18 cm/s and 189 μm at 1.81 cm/s. Other investigators who also measured fluidized nanoagglomerate sizes in the splash zone include Valverde et al. (2008a) who studied the effect of using fluidizing gases of different viscosities and Hakim et al. (2005b) who fluidized NPs at reduced pressure (with vibration) to study the effect of low pressure on the minimum fluidization velocity. While visualizing agglomerates in the splash zone seems more reliable and better than ex-situ analysis of sampled agglomerates, it is questionable whether these agglomerates are truly representative for the average bed material. Hakim et al. (2005b) argues that the method is representative since no size segregation in the bed nor a change over time of the agglomerate size was observed. While the absence of size segregation might be the case for their specific situation, it has been observed by other researchers when fluidizing nanopowder. Moreover, the dynamic nature of agglomerates makes it very well conceivable that the size and/or weight will differ with height (Quintanilla et al. 2012).

Gundogdu et al. (2007) determined the agglomerate size in the bed using X-ray microtomography; they were able to reach a spatial resolution of 400 nm. They applied this technique to fluidized beds of zinc oxide and copper oxide. They found an average agglomerate size of around 500 μm, but with a very large spread: it ranged from about 10 μm to 2 mm. Remarkably, they report an agglomerate porosity of around 50%, whereas most other authors report values as high as 98–99%. Recently, Quevedo and Pfeffer 2010 measured the size of fluidized agglomerates of both APF- and ABF-type nanopowders in-situ in conventional and assisted gas-fluidized beds using Lasentec focused beam reflectance method (FBRM) and particle vision measurement (PVM) probes. Both in-situ particle size distributions and agglomerate images of Aerosil R974 (APF type) and Aerosil 90 (ABF type) nanopowders were obtained. This was achieved by reducing the electrostatic charge in the fluidized bed by bubbling the gas through an alcohol–water solution before entering the bed. Failure to remove electrostatic charges resulted in blocking of the probe lenses and blurred images or spiky size distributions. The agglomerate size distributions showed that Aerosil R974 agglomerates are smaller and less dense than Aerosil 90 agglomerates. These observations match their respective fluidization behavior and confirm that the APF–ABF classification is dependent on both the size and density of the agglomerates. A comparison of FBRM volume weighted mean agglomerate size with that measured in the splash zone by different investigators for fluidization of Aerosil R974 is given in Table 2.

**Table 2:** Comparison of FBRM volume weighted mean agglomerate size with that measured in the splash zone by different investigators for fluidization of Aerosil R974 (Quevedo and Pfeffer 2010)

Reference	(Quevedo and Pfeffer 2010)	(Nam et al. 2004)	(Zhu et al. 2005)	(Wang et al. 2006a)	(Valverde et al. 2008a)
Conventional, in nitrogen	276 μm at 2.6 cm/s;	234 μm^a^	315 μm at 0.5 cm/s	262 μm at 1.18 cm/s;	176 μm at 1.37 cm/s
	281 μm at 3.0 cm/s			189 μm at 1.81 cm/s	
Conventional in neon	–	–	–	–	180 μm at 1.37 cm/s
Vibrated in nitrogen	–	177 μm^a^	–	–	–

*–* No measurements reported

^a^Gas velocity not reported

## Fluidization of nanopowders using external assistance methods

APF-type nanopowders are relatively easy to fluidize using aeration alone after very large and compact agglomerates (>500 μm) formed during storage are removed. To smoothly fluidize and process ABF-type nanopowders, some sort of external assistance is usually required; otherwise they show considerable gas-bypassing and significant elutriation of particles due to the required high fluidization velocity. Various assistance methods have been developed to enhance the fluidization of nanopowders. These methods include vibration, stirring, sound waves, pulsed flow, centrifugal fields, electric fields, and secondary gas flow from a microjet.

### Mechanical vibration

Nam et al. (2004) applied vertical sinusoidal vibration (accelerations up to 5.5 times the gravitational acceleration and vibration frequencies from 30 to 200 Hz) to a fluidized bed of Aerosil R974, an APF-type nanopowder. They were able to decrease the mean agglomerate size (see Table 2), increase bed expansion, and reduce the minimum fluidization velocity. They estimated the fluidizing agglomerate size, density, external porosity, and terminal velocity using a novel method originally developed by Valverde et al. (2001a) for micron size particles that combined the fractal structure of the agglomerate and the R–Z equation. Nam et al. (2004) also studied the mixing characteristics of the vibro-fluidized bed; these results will be discussed in a later section on “Mixing of fluidized nanopowders.”

Levy and Celeste (2006) studied the effects of both mechanical and acoustic vibration on the fluidization of fumed silica Aerosil 200. By adding horizontal vibrations (frequency up to 9.5 Hz), they reduced the minimum fluidization velocity, which was further reduced when adding 80 Hz acoustic vibrations. Horizontal vibration-assisted fluidization of three different Evonik-Degussa silica NPs was also studied by others (Harris 2008; Zhang and Zhao 2010) using vibration frequencies from 0 to 34 Hz. They observed APF and ABF fluidization behaviors with the transition occurring at different frequencies for each type of particle. Smooth APF-type fluidization was observed for all particles at frequencies greater than 16.7 Hz, but fluidization could not obtained in the absence of external agitation for the three silica NPs which they studied. This may be because the authors did not sieve the nanopowders to remove the very large agglomerates that formed due to storage.

### Mechanical stirring

Mechanical stirring of the fluidized bed is another way to improve fluidization of nanopowders. It can be carried out using a blade stirrer or using large magnetic particles. King et al. (2008) used a blade stirrer located in the bottom zone of the bed and report radial blending of the entire bed which prevents channeling. The blades sweep as close to the edges of the distributor plate as possible to minimize the opportunity for powder to collect along the base of the walls. According to King et al. (2008), radial stirring complements the axial flow of fluidizing gas and has shown to promote good fluidization behavior for cohesive and difficult to fluidize powders.

Yu et al. (2005) used magnetic particles excited by an external oscillating magnetic field to stir the bed; see also Pfeffer et al. (2010). The magnetic particles were large (1–2 mm) and heavy (barium ferrite) and did not fluidize along with the nanopowder, but translated and rotated at the bottom of the column just above the gas distributor. The electromagnetic field was provided by coils located outside the column at the level of the distributor. They found that magnetic stirring enhanced the fluidization of nanoagglomerates quite significantly by breaking up clusters of agglomerates and by hindering the formation of bubbles. Yu et al. (2005) were able to smoothly fluidize, without bubbles, large clusters (>500 μm) of Aerosil R974 nanopowder. This nanopowder fluidizes smoothly (APF type) when sieved below 500 μm. However, large and more compact agglomerates, greater than 500 μm that formed during storage (from about 0.5–10 mm), could not be fluidized with aeration alone even at a gas superficial velocity as high as 13.2 cm/s.

**Fig. 5:**
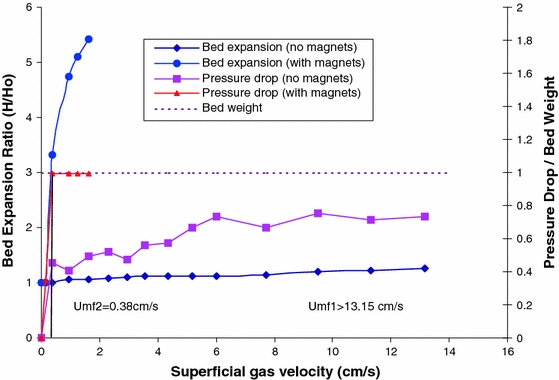
Bed expansion ratio and pressure drop for hard agglomerates with and without magnetic excitation. *Solid lines* the bed expansion ratios and *dashed lines* the pressure drops. Magnetic field intensity 140G at the center of the field, mass ratio of magnets to NPs 2:1, AC frequency 60 Hz (reprinted from Yu et al. (2005) with permission from Wiley). *Umf1* minimum fluidization velocity without magnetic excitation; *Umf2* minimum fluidization velocity with magnetic excitation

Figure 5, taken from Yu et al. (2005), shows the fluidization behavior (pressure drop and bed expansion) of the large (>500 μm) SiO_2_ NP agglomerates, with and without, magnetic excitation. Without magnetic assistance, visual observation showed that the smaller agglomerates were in motion at the top of the bed, but the larger agglomerates remained at the bottom of the bed, causing channeling of the gas flow. The bed showed almost no expansion, and the pressure drop was less than the bed weight, indicating that the entire bed was not fluidized. After turning on the external magnetic field, the large agglomerates became much smaller due to fragmentation (disruption of interparticle forces) caused by collisions with the magnetic particles, and these smaller agglomerates participated in the fluidization of the bed. After a few minutes, even at the relatively low gas velocity of 0.94 cm/s, all of the large agglomerates disappeared. The bed expanded slowly and uniformly, while the pressure drop became very close to the weight of the bed, indicating that the entire bed was fluidized. The magnetic particles were then removed, and the magnetically processed NP agglomerates were recharged back into the fluidization column, and a conventional fluidization experiment (no magnetic assistance) was performed. A very large reduction in the minimum fluidization velocity (*U*_mf_) from larger than 13.2 to 2.29 cm/s was observed, indicating that the average agglomerate size was significantly reduced.

Yu et al. (2005) also reported the average agglomerate size of sieved Aerosil R974 nanopowder less than 500 μm in size from images taken in the splash zone with and without magnetic assistance. Although the sieved nanopowder fluidized well without magnetic assistance, the difference in the measured average agglomerate size decreased from 315 to 195 μm when magnetic assistance was applied.

Yu (2005) also fluidized primary NPs of carbon black pelletized to 800 μm (Cabot Black Pearls 2000) by this method. Neither fluffy carbon black NPs nor pelletized carbon black could be fluidized with aeration alone. He showed that without magnetic excitation, the minimum fluidization velocity is 27.6 cm/s, and this high gas velocity leads to large elutriation of carbon black particles and large gas-bypassing. When magnetic excitation is applied, the minimum fluidization velocity drops to 1.93 cm/s, and this much lower gas velocity prevents elutriation and significantly reduces bubbling and gas bypass. Also, the bed expansion increased from about 1.6 to about 5 or 6 times the original bed height and the surface of the bed appears uniform.

Zeng et al. (2008) used a magnetically assisted fluidized bed similar to those described earlier (Yu 2005; Yu et al. 2005) to fluidize a mixture of APF-type SiO_2_ (20 nm) and ABF-type ZnO (20 nm) nanopowders. They found that this mixture can be fluidized stably and almost homogenously with the magnetic assistance, depending on the magnetic field intensity applied and the initial mixture content.

Quevedo et al. (2007) studied the effect of using assistance methods such as vibration and/or moving magnetic particles on the humidification and drying of fluidized Aerosil 200 and Aerosil 90 nanopowders. Moisture was added to the fluidizing gas (nitrogen) by bubbling it through water, and the moisture level in the gas was monitored on-line using humidity sensors upstream and downstream of the fluidized bed. The amount of moisture adsorbed/desorbed by the powders was obtained by integration of the time-dependant moisture concentration. The experiments were run at temperatures above the dew point, to ensure the absence of liquid water and avoid the change of particle interaction by liquid bridging. It was found that when the bed of powder is assisted during fluidization, the mass transfer between the gas and the nanopowder is much larger than when the powder is conventionally fluidized. For Aerosil 200 (APF type), the presence of large agglomerates does not affect the amount of moisture retained by the fluidized bed since they are found in small amounts. For Aerosil 90 (ABF type), large agglomerates constitute a significant fraction of the powder and they affect the adsorption of moisture due to the poor mixing between the solid and gas phases, hindering the overall adsorption of moisture by the bed of powder.

The enhancement of fluidization due to the assistance methods is reflected by the increase of moisture retained by the fluidized bed of powder during humidification and by the reduction of the time needed for the bed of powder to release the moisture trapped during drying. Vibration assistance was found to be more effective for Aerosil 200, but magnetic assistance was needed for Aerosil 90 in order to break-up the very large agglomerates formed in this ABF nanopowder. For Aerosil 90, a combination of vibration and magnetic assistance gave the best results.

### Sound waves

Zhu et al. (2004) used an external force field generated by sound in order to enhance the fluidization of APF-type Aerosil R974 fumed silica NPs. They placed a loudspeaker at the top of the bed. At sound frequencies of 50 or 100 Hz, they obtained a larger bed expansion and also a reduction in the minimum fluidization velocity. However, at frequencies greater than 200 Hz, they observed large ellipsoid-shaped bubbles which do not occur with aeration alone.

Guo et al. (2006) also fluidized fumed silica NPs under the influence of an acoustic field. At frequencies below 200 Hz, they found results similar to those of Zhu et al. (2004). Liu et al. (2007) used sound-assisted fluidization of two kinds of SiO_2_ NPs (having primary sizes of 5–10 nm); one without surface modification and the other modified with an organic compound. The acoustic field (~100 dB and 50 Hz) reduced the minimum fluidization velocity for both NPs, but the untreated silica failed to fluidize as smoothly as the surface-modified silica. Different fluidization behavior, different bed expansion, and agglomerating behavior were also observed for the two kinds of NPs, which indicate that the surface properties of NPs have a significant influence on their fluidization behavior. Similar results were previously reported (Zhu et al. 2005) when comparing the fluidization behavior of hydrophilic and hydrophobic silicas without external assistance.

Sound-assisted fluidization of silica and alumina nanopowders was also recently studied by Ammendola and Chirone (2010). As already reported by others above, they found the fluidization quality of both nanopowders to be poor without external assistance, even though some bed expansion was found. However, the application of acoustic fields of intensities above 135 dB and frequencies around 120 Hz increased the fluidization quality of both powders as indicated by ideal-like pressure drop curves, relatively high bed expansions, and the occurrence of a homogeneous regime of fluidization. A drawback of the use of sound waves produced by a loudspeaker placed at the top of the bed is that just the region close to the free surface can be excited, while larger and heavier agglomeration are mainly present at the bottom of the bed.

### Pulsed gas flow

Rahman (2009) applied pulsations to the gas flow in a fluidized bed of different nanopowders; only part of the gas flow was oscillated (i.e., there is a constant base flow). She found that the fluidization quality is significantly improved compared to steady gas flow conditions: the solids motion was enhanced, channeling was prevented, and the minimum fluidization velocity decreased. Gas phase pulsation was found to be especially effective when fluidizing ABF-type nanopowders which tend to bubble as soon as minimum fluidization conditions are reached and shows very little bed expansion when fluidized conventionally. By applying pulsation assistance, bubbles bursting at the bed surface were greatly inhibited, and bed expansion was higher than for steady flow conditions. It was also found that the minimum fluidization velocity decreased when increasing the pulsation frequency. A disadvantage is that pulsation can lead to increased elutriation. On the other hand, gas pulsation can be used effectively to improve the quality of NP fluidization without adding any internals or foreign material to the bed, such as when using magnetic-assisted fluidization.

### Centrifugal field

The use of a rotating fluidized bed to impose a centrifugal field on nanopowders has some distinct advantages over a conventional fluidized bed. The centrifugal force acting on the agglomerates allows fluidizing them at much higher gas velocities resulting in a much higher gas throughput per unit area of distributor, less entrainment of particles, and shallow beds resulting in very small bubbles and therefore very little gas-bypassing. Fumed silica, alumina, and titania nanopowders have been successfully fluidized in a rotating fluidized bed (Matsuda et al. 2004; Nakamura and Watano 2008; Quevedo et al. 2006). A smooth surface and appreciable bed expansion were obtained when using APF nanopowders, but ABF nanopowders such as Aeroxide titania P25 did not expand significantly due to bubbling.

**Fig. 6:**
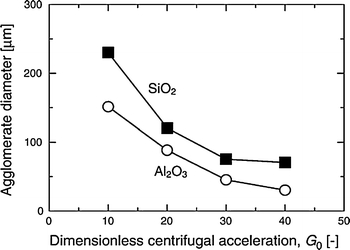
Agglomerate size of nano-particles as a function of centrifugal acceleration for a Richardson and Zaki exponent *n* = 5 (reprinted from Nakamura and Watano (2008) with permission from Elsevier)

**Fig. 7:**
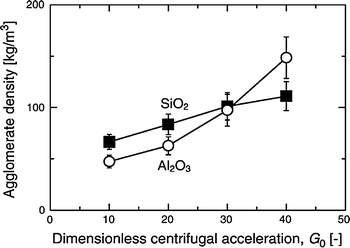
NP agglomerate density as a function of centrifugal acceleration. *Error bars* the differences with a change of *n* in a range of 4–6 (reprinted from Nakamura and Watano (2008) with permission from Elsevier)

Nakamura and Watano (2008) showed that minimum fluidization velocity increases linearly with *G*_0_ for different metal oxide nanopowders and is highest for Aeroxide titania P25 (ABF type). The fully expanded bed height is found to decrease with increasing *G*_0_ for alumina and silica nanopowders, but was difficult to measure for the ABF-type titania due to bubbling. As shown in Fig. 6, the mean agglomerate size of Aerosil R974 NPs calculated using the fractal model suggested by Valverde et al. (2001a) is reduced by a factor of as much as four for high *G*_0_ (40 times the acceleration of gravity) as compared to a conventional fluidized bed (*G*_0_ = 1). As expected the agglomerate density (Fig. 7) in an RFB is larger than that in a conventional fluidized bed and is also larger than in vibration and magnetic-assisted fluidized beds.

Matsuda et al. (2004) also studied the fluidization of a 7-nm primary particle size nanopowder in a rotating fluidized bed. They developed a model for predicting the agglomeration of NPs based on an energy balance between the energy required for disintegration of the agglomerates and the attainable energy for disintegration of the agglomerates. Experimentally, they found that the agglomerate size is reduced not only with increasing *G*_0_ as reported by Nakamura and Watano (2008), but also with long-term operation of the fluidization.

### DC and AC electric fields

Kashyap et al. (2008) studied the fluidization behavior of Tullanox 500 (an APF-type fumed silica nanopowder having a typical primary particle size with a diameter of 10 nm) in a rectangular fluidized bed with a DC electric field. Two copper sheets, acting as the two electrodes with opposite polarities, were attached to the parallel walls in the rectangular fluidized bed. Each electrode was connected to one of two high-voltage DC power supplies capable of producing up to 8 kV of DC voltage with opposite polarities, thus producing a maximum of 16 kV when connected to the electrodes. For the electrofluidization of Tullanox 500 NP agglomerates, the fluidized bed height was found to decrease rather than increase when the DC electric field was applied.

Quintanilla et al. (2008) found similar results for DC electrofluidization of Aerosil R974. The Sevilla Powder Tester (SPT) (Quintanilla et al. 2008) was utilized as the fluidization setup, and two electrodes were placed on either side of the column and were connected to a DC high-voltage source. One of the electrodes was grounded and a high voltage (up to 30 kV) was applied to the opposite electrode using a high-voltage DC supply. The application of the electric field resulted in a decrease of the height of the bed. The decrease was not reversible. After turning off the electric field, the height of the bed further decreased or remained the same, rather than return to its previous height.

The reason for the decrease in fluidization quality upon applying a DC electric field is that the NP agglomerates migrate toward the walls of the cell as seen by direct visualization using a high-speed camera (Valverde et al. 2008b). The charged nanoagglomerates feel a force *F* = *Q*·*E*, where *Q* is the charge on the agglomerates and *E* is the DC electric field strength. By this force, they are moved toward the walls of the fluidization column where they get irreversibly stuck. Thus, the fluidized bed appears to behave more like a spouted bed with most of the gas-bypassing through a central channel depleted of agglomerates, which results in the observed decrease in bed expansion.

Quintanilla et al. (2008) also studied the expanded state of the fluidized bed under the combined effects of both vertical vibration and a DC electric field (provided by electrodes surrounding the bed). When the vibration was applied to the fluidized bed, the overall solid volume fraction *ϕ* decreased (i.e., the bed height increased), and the quality of fluidization improved as was previously observed (Harris 2008; Levy and Celeste 2006; Nam et al. 2004; Valverde et al. 2001a; Zhang and Zhao 2010). As the gas velocity was increased, the reduction in *ϕ* decreased implying that the vibration has less effect on the expanded state at high velocities (velocities much greater than the minimum fluidization velocity). Experiments performed at certain vibration frequencies also showed the formation of bubbles that propagated throughout the bed which curtailed bed expansion. The formation of bubbles occurred at different frequencies, depending on both the superficial gas velocity and effective vibrational force. By varying the strengths of the external fields (vibration and electric field), it was possible to achieve an equilibrium state, which matched the expanded state of the bed under no external effects. When only vibration was applied to the fluidized bed, the quality of fluidization improved. However, when a DC electric field was applied, the bed expansion decreased dramatically, probably due to electrophoretic deposition of the particles which made them stick to the wall of the column and not participate in the fluidization.

Since the DC electric field actually decreased the NP fluidization quality, researchers recently studied the effect of applying an AC electric field (Lepek et al. 2010; Espin et al. 2009). In both studies, Aerosil R974 was used as the bed material. Espin et al. (2009) used a cylindrical column and applied a horizontal electric field (cross-flow). They found that the AC field works by agitating the charged agglomerates, for which an optimum frequency is needed in order to avoid electrophoretic deposition at the walls. This was observed at low frequencies, while at very high frequencies, agglomerates do not appear to be agitated and there is no observable effect of the field.

**Fig. 8:**
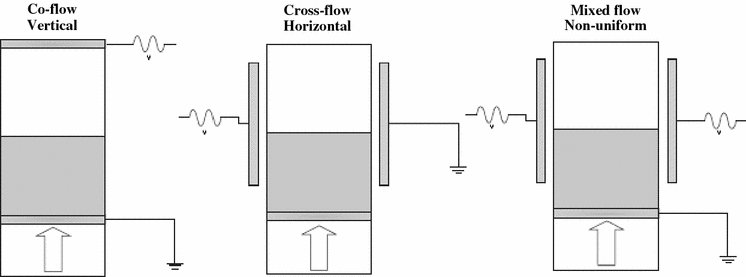
Sketches of the three different setups used in the alternating electric field enhanced fluidization: **a** co-flow electric field, **b** cross-flow electric field, **c** variable electric field (reprinted from Lepek et al. (2010) with permission from Wiley)

**Fig. 9:**
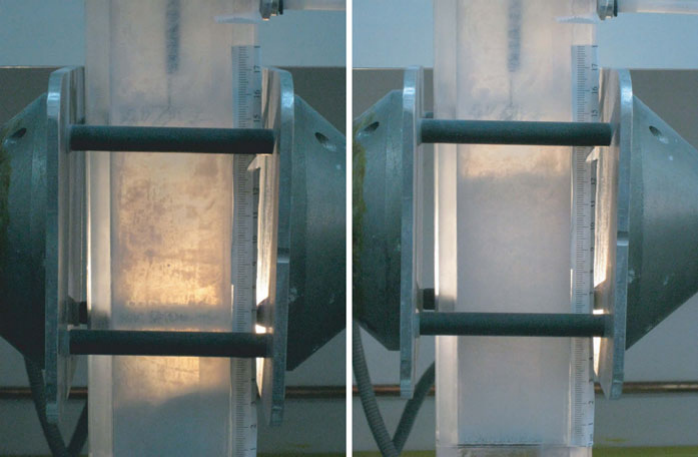
Snapshots of a fluidized bed of unsieved R974 silica before (*left*) and after (*right*) the electric field was applied (variable field configuration) (reprinted from Lepek et al. (2010) with permission from Wiley)

Lepek et al. (2010) used a rectangular fluidization cell made of polycarbonate. They applied three different electric field spatial distributions (Fig. 8): a vertical field configuration (co-flow field), a horizontal electric field configuration (cross-flow field) which is the same configuration used in Quintanilla et al. (2008) for the DC electric field experiments, and a variable field configuration (non-uniform field). The latter used the two vertical electrodes for the cross-flow held at the same high voltage and grounding the metallic distributor plate at the bottom of the fluidization cell. In the non-uniform field configuration, the highest potential difference occurred in the region between the vertical electrodes and the distributor plate (Lepek et al. 2010). Thus, the largest induced electric field is applied in this region. On the other hand, the field between the vertical electrodes is negligible for a bed height of the order of the separation between the electrodes. All of the three different alternating electric fields configurations (co-flow, cross-flow, and variable) were found to enhance bed expansion. For the co-flow electric field, the polarity of the electrodes plays a major role in the expansion behavior with the top electrode grounded arrangement producing a higher bed expansion. In the cross-flow configuration, some bed expansion occurred, but at high velocities, some of the powder was elutriated. The most effective technique to assist fluidization was the application of the non-uniform alternating electric field (see Fig. 9), which was weak in the vicinity of the free surface but strong close to the bottom of the bed.

Due to the wide size and weight distribution of the NP agglomerates—especially with unsieved nanopowder—a conventional fluidized bed is highly stratified: larger and heavier agglomerates will sink to the bottom of the bed, and smaller and light agglomerates will be suspended close to the free surface. These light agglomerates are easily elutriated if the gas flow is increased to mobilize the heavier agglomerates. The alternating non-uniform electric field strongly agitates the heavier agglomerates, which destabilizes the development of gas channels close to the distributor, thus enhancing fluidization. Furthermore, the variable field has almost no effect on the light agglomerates at the top of the bed, thus avoiding excessive elutriation. This arrangement’s greatest advantage is helping to assist in the fluidization of unsieved nanopowder, which has a wide agglomerate size distribution range. Using this technique, the powder does not have to undergo a pre-treating sieving process, which has been critical to most previous fluidization studies of R974 silica.

### Secondary flow using microjets

Secondary flows in the form of jets to fluidize micron-sized particles have been widely studied. Research has been done with jets pointing upwards, downwards, or horizontally, typically with nozzle sizes of the order of millimeters. These studies have shown that when properly designed and at high gas velocities, jets enhance fluidization by promoting turbulent mixing. Quevedo et al. (2010) and Pfeffer et al. (2008) have recently described a new method for enhancing the fluidization of agglomerates of NPs based on the use of microjets produced by micro-nozzles (diameters ranging from 127 to 508 μm) pointing downwards at close distance to the air distributor. Micro-nozzles pointing upwards also work, but there is some powder between the distributor and the nozzles that may not participate in the fluidization. In their experiments, nitrogen was used as the fluidizing gas. A low-pressure line was used to feed gas to the column through the distributor plate which is considered the primary flow, and a medium pressure line (about 8 bar) supplies gas to the micro-nozzle or nozzles and is the secondary flow. Part of the primary flow was bubbled through a tank containing a dilute ethanol−water solution which substantially reduces electrostatic effects in the fluidized bed caused by triboelectrification (Pfeffer and Quevedo 2011). The nanopowders used were different metal oxides (silicas, alumina, and titania) supplied by Evonik-Degussa. These powders were sieved to remove clusters of agglomerates larger than either 500 or 850 μm that formed during transportation and storage.

**Fig. 10:**
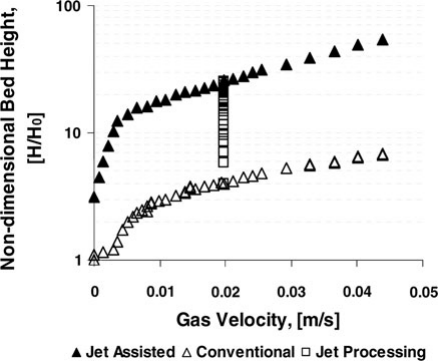
Comparison of the non-dimensional fluidized bed height as a function of gas velocity for conventional and microjet-assisted fluidization of Aerosil R974 (reprinted from Quevedo et al. (2010) with permission from Wiley)

According to Quevedo et al. (2010), the use of a micro-nozzle or multiple micro-nozzles as a secondary flow produced a microjet with sufficient velocity (hundreds of meters per second) and shear to break-up large nanoagglomerates, prevent channeling, curtail bubbling, and promote liquid-like fluidization. For example, Aerosil R974, an APF-type nanopowder, expanded up to 50 times its original bed height after the powder was processed by the microjet for about 20 min; without jet assistance, the maximum bed expansion was about 6 times (see Fig. 10).

**Fig. 11:**
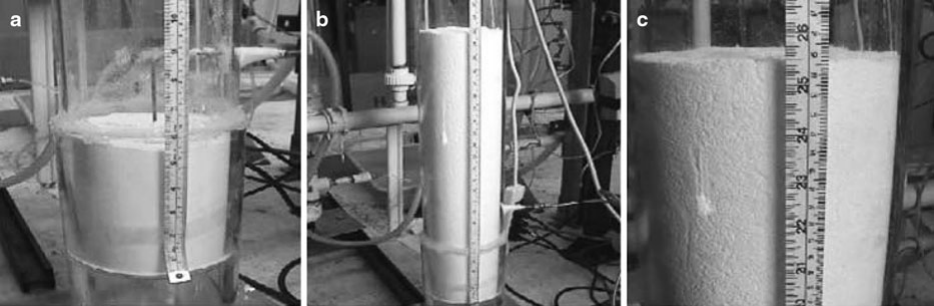
Images corresponding to the fluidization of Aeroxide TiO_2_ P25 in a 5-inch (12.7 cm) ID column. **a** Initial bed height, **b** maximum bed height when fluidized with microjet assistance, and **c** close-up of the fluidized bed surface. The fluidized bed expanded from 5.5 inches (14.0 cm) to 25.5 inches (64.8 cm), and the surface of the bed shows no bubbles (reprinted from Quevedo et al. (2010) with permission from Wiley)

Microjet assistance also allows for the conversion of ABF-type behavior into APF-type behavior. Without microjet assistance, a maximum bed expansion of about 2.5 times the initial bed height is obtained for Aerosil 90, 1.75 for Aeroxide Alu C, and only 1.25 for Aeroxide TiO_2_ P25; the latter is one of the most difficult metal oxide nanopowders to fluidize. For these nanopowders, when the superficial gas velocity is increased above a certain value, i.e., the minimum bubbling velocity (*U*_mb_), the bed does not expand further and the bed height remains constant. As a result of applying the microjet(s), fluidized bed expansion of ABF nanopowders is increased 13–15 times for A90 and Alu C, and 5–6 times for TiO_2_ (see Fig. 11). The fluidization is much smoother and more homogeneous (APF-like), there is very little, if any, elutriation, and the onset of bubbling is also delayed due to the better dispersion of the powder in the gas phase. Microjet-assisted NP fluidization was also found to improve solids motion and prevent powder packing in an internal (Quevedo et al. 2010) and can be easily scaled-up by adding additional micro-nozzles.

King et al. (2009) also used microjet-assisted NP fluidization in their atomic layer deposition (ALD) experiments in a glass fluidized bed reactor (FBR) at a pressure around 1 mbar and at temperatures between 100 and 500 °C. ALD is a gas-phase reactive process by which nanoscale functional layers can be chemically bonded to the surfaces of fine particles (see also the section “Applications and challenges”). Nozzle diameter, pressure, and relative flow rates were studied at a variety of conditions to optimize NP fluidization behavior in the presence of reactive precursors. In a new ALD study to coat ZnO onto TiO_2_ NPs, King et al. (2010) used a microjet-assisted FBR with isopropyl alcohol-based (instead of water) ALD to remove undesirable electrostatic effects as suggested by Pfeffer and Quevedo (2011). They also used a rotating tube suspended in the center of the reactor to which three micro-nozzles (two upward facing and one downward facing) were attached. This configuration, along with the alcohol-based ALD process, increased the dense phase to bubble phase ratio in the FBR to 89:11 from 55:45 using conventional water-based ALD.

## Mixing of fluidized nanopowders

Some studies have been devoted to the mixing of fluidized nanopowders, both to the mixing of the agglomerates as well as the mixing inside agglomerates (i.e., exchanges of material between agglomerates). Nam et al. (2004) studied the mixing characteristics of the vibro-fluidized bed of NPs by dying some of their nanosilica blue to act as a tracer. They found very good mixing after 2 min of fluidization (the entire column of particles turned blue). Huang et al. (2008) studied the mixing of silica R972 by adding less that 5% phosphor particles with a diameter of 3.7 μm to the nanopowder. By mixing the materials well, composite agglomerates were formed, and the phosphor particles were used as tracers. By giving a light pulse and using a photosensitive detector, the mixing rate was determined. Huang et al. (2008) showed that the mixing rate was much lower than for a bed of FCC particles: both the radial and the axial dispersion coefficients were two orders of magnitude lower.

Nam et al. (2004) also reported some preliminary testing with mixing of different materials (nano-silica with nano-titania and nano-molybdenum oxide) with SEM–EDX (scanning electron microscope–energy dispersive using X-ray analysis). They observed proper mixing of the agglomerates, but could not determine whether the agglomerates retained their integrity during fluidization or whether they broke and formed again rapidly. Hakim et al. (2005b) colored two batches of Aerosil OX-50 silica NPs with red and green dye, and put the two batches together with an uncolored (white) batch of the same material in a fluidized bed column. The powders were fluidized together for 1 h under mechanical vibration, and a sample of the resulting powder was analyzed under a light microscope. They observed agglomerates containing all three colors, indicating that the initial agglomerates broke apart and reformed into new complex agglomerates. This result offers qualitative evidence of the dynamic agglomeration of pre-existing NP agglomerates during fluidization, although Hakim et al. (2005b) did not report the scale of the mixing.

**Fig. 12:**
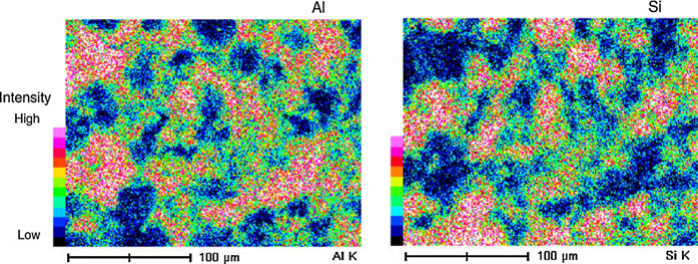
Element mapping images of film surface of mixing sample (*G*_0_ = 40; *U*_0_/Umf = 1.5; SEM magnification was 1,000 times; mixing time was 6 min) (reprinted from Nakamura and Watano (2008) with permission from Elsevier)

Nakamura and Watano (2008) performed more detailed mixing studies of different NPs, nanosilica, and nanoalumina, in a rotating fluidized bed. They also obtained good mixing, but the mixing occurred at a scale of about 50 μm as shown in the SEM–EDX images (see Fig. 12). Apparently, parts of the agglomerates are exchanged, but the mixing did not take place down to the scale of individual NPs. This could partly be explained by the fact that the used NPs are produced by flame synthesis and might have formed sintered networks (also called sub-agglomerates), but such networks are typically not larger that 1 μm. Apparently, also Van der Waals forces and possibly capillary forces play a role (see the section “Forces between NPs”) in keeping the sub-agglomerates together at a scale around 50 μm.

Ammendola and Chirone (2010) applied SEM–EDX analysis to samples of a sound-assisted NP fluidized bed of initially unmixed alumina and copper oxide. They concluded from color tracing that mixing of the agglomerates required just a few minutes, while mixing inside the agglomerates (e.g., exchange of material at the μm scale) required 80–150 min. Quevedo et al. (2010) performed NP fluidization experiments with alumina and iron oxide nanopowders, and studied powder samples using TEM–EELS (transmission electron microscopy–electron energy-loss spectroscopy). This enabled them to investigate the mixing behavior of the two nanopowders at the nanoscale. They found that for conventional fluidization mixing occurred only at the microscale; no mixing at the nanoscale took place. However, a powder sample after microjet processing was completely mixed and agglomerates had indeed exchanged individual NPs. This indicated that microjets can promote nanoscale mixing, while other assistance methods only seem to yield micro-scale mixing (i.e., exchange of sub-agglomerates).

## Modeling of NP fluidized beds

### The size of NP agglomerates

A number of semi-empirical models can be found in the literature aimed to predict agglomerate size in NP fluidized beds. Chaouki et al. (1985) proposed that NP agglomerates in the fluidized bed are clusters of the fixed bed existing previous to fluidization. The size of the agglomerates can then be inferred from the balance between the attractive van der Waals force between particles and the agglomerate weight, which should be equal to the drag force on the agglomerate at minimum fluidization. Morooka et al. (1988) proposed an energy balance model for estimating agglomerate size, in which the energy generated by laminar shear plus the kinetic energy of the agglomerate was equated to the energy required to break the agglomerate.

Iwadate and Horio (1998) presented a model to predict the agglomerate size in a bubbling bed. In their model, they postulated that the adhesive force between agglomerates was balanced by the expansion force caused by bubbles, yet this model cannot be applied to non-bubbling fluidization. Zhou and Li (1999) proposed an equation in which the joint action of the drag and collision forces is balanced by the gravitational and cohesive force. Nevertheless this approach is only valid at high Reynolds number (turbulent flow), while typical values of the Reynolds number around the agglomerate in fluidized beds of NPs are small (Zhu et al. 2005). Mawatari et al. (2003) wrote a force balance between the van der Waals attractive force between agglomerates and the separation forces, including gravity, drag force, and vibration if present. Matsuda et al. (2004) have proposed an energy balance equation based on the assumption that there exists an attainable energy for disintegration of agglomerates proportional to a power law of the effective acceleration. The exponent of this power law was fitted to experimental results.

An inconvenience of these semi-empirical models for estimation of agglomerate sizes is that they require input of several experimental observations, which are unknown a priori.

Data on the minimum fluidization gas velocity are needed in the Morooka et al. (1988) model. Bed porosity data are required in the equation derived from the models of Matsuda et al. (2004) and Mawatari et al. (2003), the latter one requiring also measurements of the minimum velocity for channel breakage. The relative agglomerate velocity appears in the predictive equation proposed by Zhou and Li (1999). Other fluidized bed data necessary in the above described models are bed void fraction, bubble size, particle pressure in the bubbling bed, and coordination number of the agglomerates at minimum fluidization. For a detailed review of these models, the interested reader may see the review by Yang (2005).

Castellanos et al. (2005) presented a predictive equation to estimate agglomerate size originally derived to estimate the size of agglomerates of micron-sized particles in a fluidized bed. This equation was derived from a general model that considers the limit of mechanical stability of tenuous objects (Kantor and Witten 1984). In the fluidized state, micron-sized primary particles agglomerate due to the action of the interparticle attractive force *F*_0_, which in most cases is due to van der Waals interaction (Castellanos 2005). The weight force of the agglomerate, which acts uniformly through the agglomerate body, is compensated by the hydrodynamic friction from the surrounding gas, which acts mainly at its surface due to the flow screening effect. As the agglomerate grows in size, the local shear force on a particle attached at the outer layer of the agglomerate was estimated as $F_{\text{s}} \approx W_{\text{p}} \,k_{\text{a}}^{{(D_{\text{a}} + 2)}}$, where *W*_p_ is the particle weight, *k*_a_ is the ratio of the agglomerate size to particle size, and *D*_a_ is the fractal dimension of the agglomerate (Castellanos et al. 2005). Particles would continue to adhere to the agglomerate as long as the interparticle attractive force *F*_0_ is larger than *F*_s_. Thus, the balance *F*_s_ = *F*_0_ served to find an equation to predict the agglomerate size limit:

4 $$ k_{\text{a}} \approx B{\text{o}}_{\text{g}}^{{\frac{1}{{D_{\text{a}} + 2}}}} $$

where *B*o_g_ is the granular Bond number defined as the ratio of interparticle attractive force *F*_0_ to particle weight *W*_p_.

This model was later adapted by Valverde and Castellanos (2007a) to NP fluidization by considering NP simple agglomerates, which exist before fluidization, as effective particles undergoing agglomeration due to attractive forces between them in the NP fluidized bed. Thus Eq. 4 was adapted to calculate the complex agglomerate size *d* ^**^:

5 $$ d^{ * * } \approx d^{ * } \left( {\frac{F}{{W^{ * } }}} \right)^{{\frac{1}{{D_{\text{a}} + 2}}}} $$

where *d* ^*^ is the size of the simple agglomerates, *F* is the attractive force between these simple agglomerates, *W* ^*^ is their weight, and *D*_a_ is the fractal dimension of the complex agglomerates. According to statistical analysis on TEM images (Sánchez-López and Fernández 2000) and other indirect measurements (Nam et al. 2004; Wang et al. 2006b), *D*_a_ is close to 2.5. SEM images show that *d* ^*^ is, generally, of the order of tens of microns. A typical value of *F* is 10 nN when it is assumed that the main source of attraction between the simple agglomerates is the van der Waals interaction. This value may increase if particles are hydrophilic and the fluidized air is not dried, which leads to the formation of capillary bridges between the agglomerates (Valverde and Castellanos 2007a). *W* ^*^can be calculated as $W^{ * } = (d^{ * } /d_{\text{p}} )^{{D_{\text{a}} }} W_{\text{p}}$, where *d*_p_ is the size of primary NPs and *W*_p_ their weight.

Results predicted from Eq. 5 yielded agglomerate sizes of the order of hundreds of microns. These results were compared with experimental data reported in the literature on a variety of conditions (particle size and density, particle surface hydrophobicity, use of fluidization assistance techniques, etc.). Good agreement was generally found (Valverde and Castellanos 2007a). Moreover, according to Eq. 5, the physical properties of the fluidizing gas, such as gas viscosity and density, should not affect agglomerate size. This was confirmed in a work in which the mean agglomerate size was measured directly from laser-based planar imaging and indirectly derived from bed expansion data for fluidization of titania and silica with nitrogen and neon (Valverde et al. 2008a).

### The role of effective acceleration on agglomerate size in the NP fluidized bed

The effective acceleration *g*_ef_ in the fluidized bed can be increased by means of a centrifugal fluidized bed setup. The increase of the effective acceleration *g*_ef_ would cause an increase of the effective weight of the particles, which would decrease the Granular Bond number and therefore the size of the agglomerates according to Eq. 5.

Matsuda et al. (2004) carried out an extensive series of centrifugal fluidized bed experiments on titania NPs. The agglomerate size was inferred from the fit of measurements of the minimum fluidization velocity to empirical correlations with the agglomerate Archimedes and Reynolds numbers. The results indicated a decrease of agglomerate size as *g*_ef_ was increased, in good agreement with the values predicted by Eq. 5 (Valverde and Castellanos 2007a).

Other techniques to change the effective acceleration field in a NP fluidized bed and, thus, to modify agglomerate size is to apply an external source of energy such as vibration (Quintanilla et al. 2008; Nam et al. 2004) or an alternating electric field (Lepek et al. 2010). In the case of vertical vibration, the root-mean-squared effective acceleration is increased up to *g*_ef_~*g* Λ (Valverde and Castellanos 2006a), where

6 $$ \Uplambda = 1 + \frac{{A\omega^{2} }}{{g_{{}} }} $$

here *A* is the vibration amplitude, *ω* = 2π*f*, where *f* is the vibration frequency, and *g* = 9.81 m/s^2^ is the gravitational acceleration. The consequent decrease of agglomerate size according to Eq. 5, with W^*^ multiplied by Λ, would then explain the increase of fluidized bed expansion observed experimentally (Nam et al. 2004; Quintanilla et al. 2008; Valverde and Castellanos 2008).

The effective acceleration can be also increased by means of application of an alternating electric field to the fluidized bed. Since NP agglomerates are generally charged due to triboelectric charging, an externally applied oscillating electric field agitates the agglomerates in a non-invasive way. This gives rise to an additional shear force in order to balance the electrical force on the agglomerates. In the case of a horizontal electric field, the root mean square effective acceleration would be increased by a factor (Espin et al. 2009):

7 $$ \Uplambda = \sqrt {1 + \left( {\frac{{Q^{ * * } E_{\text{rms}} }}{{W^{ * * } }}} \right)^{2} } $$

where *Q* ^**^ and *W* ^**^ are the electrical charge and weight, respectively, of the complex agglomerates, and *E*_rms_ is the root-mean-square of the alternating electric field strength. Again, the predicted decrease of agglomerate size according to Eq. 5 would explain the increase in bed expansion observed for NP fluidized beds excited by alternating electric fields (Espin et al. 2009). Nevertheless, the possible influence of the increased drag on particles oscillating with respect to the surrounding fluid, which is well known to occur in liquid suspensions, should be also addressed in future investigations (Chan et al. 1972).

A relevant result also predicted by Eq. 5, but to our knowledge unobserved experimentally, is that the agglomerate size increases as the effective acceleration is decreased. Accordingly, gas fluidization of NPs at microgravity conditions would lead to the formation of extremely porous beds as seen in liquid suspensions, where agglomerate size is limited by thermal agitation.

### A modified R–Z equation for NP fluidized bed expansion

The R*–*Z phenomenological equation is widely accepted to correlate the superficial fluidizing velocity *v*_f_ and the particle volume fraction *ϕ* of uniformly fluidized beds (Richardson and Zaki 1954):

8 $$ \frac{{v_{\text{f}} }}{{v_{\text{p0}} }} = \left( {1 - \phi } \right)^{n} $$

*v*_*p*0_ is the Stokes settling velocity of a single particle at low particle Reynolds number

9 $$ v_{\text{p0}} = \frac{1}{18}\frac{{\left( {\rho_{\text{p}} - \rho_{\text{f}} } \right){\kern 1pt} {\kern 1pt} g{\kern 1pt} d_{\text{p}}^{2} }}{\mu } $$

where *ρ*_p_ is the particle density, *ρ*_f_ is the fluid density, *d*_p_ is the particle size, and *μ* is the viscosity of the fluid. The exponent *n* in Eq. 8 is an empirical parameter. Richardson and Zaki (1954) reported in their pioneer experimental work *n* = 4*.*65 in the small particle Reynolds number (Re_*t*_) regime, while *n* decreased as Re_*t*_ increased. A theoretical derivation by Batchelor and Wen (1982) for Re_*t*_ < 0*.*1 using a renormalization method led to the equation *v*_f_/*v*_p0_ ≈ 1 − 5*.*6*ϕ*, which conforms to the dilute limit of the R–Z equation for *n* = 5*.*6.

Originally, the R–Z equation was derived for fluidization of noncohesive coarse beads (of size *d*_p_ > ~50 μm) fluidized by liquids, which normally exhibit uniform fluidization. It has been shown that a modified version is also a useful correlation for uniform gas-fluidized beds of agglomerated fine and ultrafine particles (Nam et al. 2004; Valverde et al. 2001b). In this case, particle agglomeration changes the internal flow length scale, which turns out to be determined by the agglomerate size instead of the individual particle size. Thus, in the case of NP fluidized beds, the velocity scale in the R–Z equation for fluidized beds of agglomerates should be changed to the terminal settling velocity of the fluidizing units *v* ^**^, namely the agglomerates.

According to this argument, Wang et al. (2002) fitted their experimental data on NP fluidized beds to the modified equation

10 $$ \frac{{v_{\text{g}} }}{{v^{ * * } }} = \left( {1 - \phi } \right)^{n} $$

By considering *v* ^**^and *n* as fitting parameters, writing $v^{ * * } = (1/18)\rho^{ * * } gd^{ * * } /\mu$, and assuming that the agglomerate density *ρ* ^**^ could be approximated by the powder bulk density *ρ*_b_, Wang et al. inferred the agglomerated sizes in fluidized beds of several nanopowders. A similar approach was adopted by Jung and Gidaspow (2002), who used the agglomerate size obtained in this way as an input to an elaborate simulation aimed to describe the sedimentation of the bed.

Since *n* was considered as a fitting parameter, Wang et al. (2002) obtained values of *n* as low as 3, which should correspond to turbulent conditions (Richardson and Zaki 1954), yet the Reynolds number in fluidized beds of NPs is typically smaller than 0.1 (Zhu et al. 2005). It may be argued that, since NP fluidized beds operate in the low Reynolds number regime, the R–Z exponent cannot be used as a free fitting parameter, but instead it must be fixed to a value around *n* ≈ 5 corresponding to the viscous limit (Batchelor and Wen 1982).

Equation 10 has been further improved in order to take into account the effective screening of the gas flow by the agglomerates. Valverde et al. (2001b) assumed that agglomerates are approximately spherical and that the agglomerate hydrodynamic radius can be approximated to its gyration radius. As estimated by Zhu et al. (2005), the error in assuming that NP agglomerates behave as impermeable particles for the purposes of hydrodynamic analysis is small. Thus the agglomerate volume fraction *ϕ* ^**^ was used instead of the particle volume fraction *ϕ* in this modified approach:

11 $$ \frac{{v_{\text{s}} }}{{v^{**} }} = \left( {1 - \phi^{**} } \right)^{n} $$

*ϕ* ^**^ being the volume fraction of the complex agglomerates in the NP fluidized bed.

It is worth reminding that the agglomerates observed in NP fluidized beds may show an intricate hierarchical structure (Wang et al. 2002), wherein individual NPs first linking into a three-dimensional netlike structure (sub-agglomerates), which then coalesce into the simple agglomerates. According to Wang et al. (2002), these simple agglomerates aggregate into complex agglomerates when the bed is fluidized. Taking into account this multi-stage agglomerate structure (see the section “The fractal morphology of NP agglomerates”), Eq. 11 has been rewritten as (Valverde and Castellanos 2006b):

12 $$ \frac{{v_{\text{g}} }}{{v_{\text{p0}} }} = \frac{{N_{ 0} }}{{k_{ 0} }}\frac{N}{{k^{{}} }}\frac{{N^{*} }}{{k^{*} }}\left( {1 - \frac{{k_{ 0}^{3} }}{{N_{ 0} }}\frac{{k^{3} }}{N}\frac{{(k^{ * } )^{3} }}{{N^{ * } }}\phi } \right)^{n} $$

where *N*_*0*_ is the number of individual NPs aggregated in the so-called sub-agglomerates of size *d*_0_ and *k*_0_ = *d*_0_ */d*_p_ is the relative size of these sub-agglomerates (related by a fractal dimension *D*_0_ = ln *N*_0_ */* ln *k*_0_). *N* is the number of sub-agglomerates aggregated in the so-called simple agglomerates of size *d* ^*^ and *k* = *d* ^*^ */d*_0_ is the relative size of these simple agglomerates (related by a fractal dimension *D* = ln *N/* ln *k*). Finally, *N* ^*^ is the number of simple agglomerates (existing before fluidization) that aggregate in the fluidized bed to form the so-called complex agglomerates of size *d* ^**^ and *k* ^*^ = *d* ^**^ */d* ^*^ is the relative size of these complex agglomerates (related by a fractal dimension *D* ^*^ = ln *N* ^*^/ln *k* ^*^). Likewise, the predictive equation to estimate agglomerate size (Eq. 4) can be further elaborated to take into account this multi-step agglomeration process (Valverde and Castellanos 2008).

Equation 11 allows us to incorporate in the model any additional knowledge about the multiple agglomeration steps that originate the complex agglomerates. It might well happen that the fractal dimension of the simple agglomerates *D* = ln *N/*ln *k* is not the same as the fractal dimension of the complex agglomerates *D* ^*^ = ln *N* ^*^/ln *k* ^*^, or the fractal dimension of the sub-agglomerates *D*_0_ = ln *N*_0_/ln *k*_0_. That will depend on the agglomeration mechanism of NPs in the nanopowder synthesis process. In that case, the global fractal dimension *D*_a_ = ln *N*_a_/ln *k*_a_ of the complex agglomerate, where *N*_a_ = *N* ^*^ *N* *N*_*0*_ and *k*_a_ = *k* ^*^ *k* *k*_*0*_ *,* would not be well defined. By assuming that the global fractal dimension definition is valid (*D*_0_ = *D* = *D* ^*^ = *D*_a_), Eq. 12 can be rewritten as

13 $$ \frac{{v_{\text{g}} }}{{v_{\text{p0}} }} = k_{\text{a}}^{{D_{\text{a}} - 1}} \left( {1 - k_{\text{a}}^{{3 - D_{\text{a}} }} \phi } \right)^{n} $$

where $$ k_{\text{a}} = d^{ * * } /d_{\text{p}} $$ *.*

Equation 13 has been employed to estimate the agglomerate size by fitting it to experimental results on bed expansion and sedimentation, yielding results in good agreement with direct observations by means of laser-based planar imaging (Nam et al. 2004; Valverde and Castellanos 2007a; Zhu et al. 2005; Wang et al. 2006a). In close analogy with gas-fluidized beds of micron-sized particles, the fractal dimension *D*_a_ of the complex agglomerates obtained from fitting turns to be close to 2.5. An increase of this value is observed when the quality of fluidization decreases. This indicates that there is a correlation between the higher density of agglomerates (higher values of *D*_a_) and the worsening of fluidization quality.

### The size of gas bubbles in NP fluidized beds

Having an estimation of the maximum size of stable gas bubbles (*D*_b_) in NP fluidized beds will give us an idea of the type of fluidization expected. Using a criterion originally derived by Harrison et al. (1961), it has been hypothesized that gas bubbles in NP fluidized beds are no longer stable if their rising velocity exceeds the terminal settling velocity of the complex agglomerates (Valverde et al. 2008a), which leads to the simple equation

14 $$ \frac{{D_{\text{b}} }}{{d^{**} }} \approx \frac{1}{160}\frac{{\rho_{\text{p}}^{3} {\kern 1pt} g{\kern 1pt} d_{\text{p}}^{3} }}{{\mu^{2} }}{\kern 1pt} \,k_{\text{a}}^{{2D_{\text{a}} - 3}} $$

for the ratio of maximum bubble size *D*_b_ to complex -agglomerate size in NP fluidized beds. Here *k*_a_ can be calculated from Eq. 4 and it may be assumed $D_{\text{a}} \approx 2.5$. Following the original criterion by Harrison et al. (1961), this ratio is directly correlated to the type of fluidization to be expected. Thus, if $D_{\text{b}} /d^{ * * } < 1$, the powder would exhibit APF fluidization behavior, characterized by large bed expansion and the absence of visible gas bubbles. On the other hand, a value $D_{\text{b}} /d^{ * * } > 10$ means that stable gas bubbles of macroscopic size are likely to be developed. In this case, ABF behavior, characterized by poor expansion and presence of large bubbles, is to be expected. For intermediate cases, a transition from APF to ABF behavior would occur as the gas velocity is increased.

Using Eq. 14, it was estimated, for example, $D_{\text{b}} /d^{ * * } \approx 0.4$ for fluidization of R974 silica nanopowder (Valverde and Castellanos 2007a), which led to predict full suppression of bubbles for these nanopowder as experimentally observed (Zhu et al. 2005). On the other hand, it was estimated $D_{\text{b}} /d^{ * * } \approx 3.4$ for titania P25 nanopowder (Valverde and Castellanos 2007a) which predicts, for these nanopowders, a transition to bubbling fluidization as the gas velocity is increased, in agreement with experimental observations (Zhu et al. 2005).

The use of Eq. 14, along with a modified Wallis criterion to predict the onset of bubbling instability for fluidized agglomerates, allowed for the construction of the modified Geldart’s diagram shown in Fig. 4 (Valverde and Castellanos 2007b). In the case of fluidization of nanopowders, particle size and density must be interpreted in this diagram as the size and density of the simple agglomerates existing before fluidization, which behave as effective particles when fluidized and agglomerate to form complex agglomerates. The typical density and size of these simple agglomerates for silica nanopowder are 50 kg/m^3^ and 30 μm, respectively (Valverde et al. 2008a; Zhu et al. 2005), which according to Fig. 4 would give SFE behavior (or APF in different terminology) in agreement with experimental observations. Titania nanopowders have denser simple agglomerates (density above 100 kg/m^3^), which would shift the fluidization behavior of this nanopowder to SFB (or ABF in different terminology) as seen experimentally (Valverde and Castellanos 2007b; Zhu et al. 2005).

### Computational fluid dynamics modeling of NP fluidization

Computational fluid dynamics (CFD) is routinely applied in industry to help engineering design and has also become a relevant subject of research in multiphase systems, including fluidization. Reliable simulation tools can provide valuable insights into particle flow processes and, as a result, accelerate the achievement of substantial process improvements. The challenge in modeling particulate processes lies in understanding the wide range of physical length and time scales. In order to justify a CFD study of NP fluidized beds, it is particularly relevant to begin with a proper formulation of the averaged equations and closure relations. Thus, a fundamental problem is to write down the equations that are to be solved, especially when the size of agglomerates is a dynamic variable. Usually the closure relations when formulating the basic fluid mechanics equations of fluidized beds are formulated on the basis of rough assumptions since the interpretation of empirical data from engineering studies is difficult. A valuable contribution for the success of CFD simulations of NP fluidized beds would be thus experimental results obtained either at macroscopic, mesoscopic, or microscopic scales.

A main difficulty of CFD studies is that the fluidizing units in NP fluidized beds (i.e., the complex agglomerates) are continuously undergoing a dynamic process of formation and disruption. In spite of this fundamental difficulty, some attempts have been performed to interpret experimental results on NP fluidization by means of CFD. In these works, this problem is typically circumvented by assuming a fixed agglomerate size and density, to be inferred from experimental measurements.

Jung and Gidaspow (2002) simulated the settling of a NP fluidized bed using an Eulerian−Eulerian (two-fluid) hydrodynamic model. The input into the model was a measured solids stress modulus and an agglomerate size determined from the settling curves. An interesting conclusion from their work was that the simulation results predicted nonbubbling fluidization for the NP agglomerates, while the same CFD code predicted bubbling for Geldart B particles as observed experimentally. Furthermore, the simulation results were in close agreement with the observed sedimentation velocity in the NP fluidized bed when the gas flow supply was turned off.

Wang et al. (2007a) worked on a two-fluid model based also on the solid stress modulus model developed by (Jung and Gidaspow 2002) and a drag force model proposed by Wang et al. (2002). Averaged solids concentration and particle velocity distributions were computed showing a circulation pattern of the NP agglomerates in a nonbubbling fluidized bed. An interesting result of the simulations was the stratification of solids concentration, with the highest solids concentration in the bottom of the bed. The simulation results showed reasonable agreement with experimental results reported by Jung and Gidaspow (2002). Huilin et al. (2010) used an Eulerian−Eulerian model, combined with an agglomerate-based approach. As proposed by Van Wachem and Sasic (2008), the agglomerate properties used in the simulations are estimated from a force balance, taking into account drag, collision, gravity, and Van der Waals interaction. Huilin et al. (2010) show that this leads to agglomerate sizes that are in good agreement with experimental findings.

An alternative approach to the Euler−Euler simulations is Euler–Langrange simulations. In CFD models of the latter type, the gas phase is treated as continuous and the particles are modeled individually by a discrete element model (DEM). In the case of NPs, the discrete elements are the agglomerates rather than the individual NPs (Wang et al. 2008). The agglomerate motion is calculated by integrating Newton’s law of motion and the fluid is modeled by approximating the Navier−Stokes equations in a finite volume discretized framework. Agglomerate–agglomerate interactions are calculated using the soft-sphere approach, which enables for multiple collisions occurring frequently in a dense fluidized bed. In this approach, it is assumed that when the spheres collide, they deform elastically and suffer a repulsive force of strength proportional to the magnitude of the overlap. To prevent excessively large computational times, these simulations are limited to 2D (Wang et al. 2008) or pseudo 2D (van Ommen et al. 2010a) geometries. These simulations assume a constant agglomerate size (i.e., agglomerate breakage is not considered).

Wang et al. (2008) showed by simulations that the stability analysis of Foscolo and Gibilaro (1987)—originally developed for conventional particles—is useful for predicting the transition from particulate to bubbling fluidization. van Ommen et al. (2010a) studied the high-velocity microjet technique for enhancing nanopowder fluidization. Their simulations suggested that the main cause for agglomerate size reduction and bed height increase found in microjet experiments is not the shear on the agglomerates, but rather agglomerate–agglomerate collisions: these give much larger forces on the agglomerates in the simulations.

As said above, a central problem of the current state of the art in CFD modeling on NP fluidization is that agglomerates have to be treated as rigid spheres of fixed size and density. Since complex agglomerates are formed during fluidization, experimental data have to be an input for carrying out the simulations. Fully predictive simulations to be performed in future works should allow for agglomerate size to be an output of the simulation results. A possible strategy would be to incorporate Eqs. 3 and 4 into the models. In the case that the bed is externally excited, an effective acceleration can be incorporated into the model as it has been described in the cases of vibration and AC electric field (Eqs. 5 and 6). The input parameters would be in this way primary parameters known a priori such as simple agglomerate size (to be measured by means of SEM), particle density and size, and interparticle attractive force. This approach would be useful for evaluating the effect of external fields used to assist fluidization thus helping to optimize their application in practical situations. A remaining issue would be to properly model the collisions between agglomerates that may lead to agglomerate breakage as inferred from the work of van Ommen et al. (2010a) in the case of the microjet assistance technique.

## Applications and challenges

Currently, fluidization of nanopowders is only applied in a limited number of commercial processes. The two most important large-scale processes involving fluidization of nanopowders are the production of fumed metal oxides and carbon black (Flesch et al. 2008; Voll and Kleinschmit 2000). Fumed metal oxides are nanopowders which are industrially produced in flame reactors at high temperature. In the case of fumed silica, a chlorosilane vapor (SiCl_4_) is mixed with air and hydrogen, and hydrolysis takes place well above 1,000 °C. Fumed silica is used in the silicone industry to provide the desired rheology and mechanical strength in silicone adhesives and silicone rubbers, and as filler in paints, coatings, printing inks, adhesives, and unsaturated polyester resins. Fumed alumina is used to treat ink jet paper for improved ink absorbance, and fumed titania is used in cosmetic applications such as sunscreens. In the manufacture of fumed metal oxides, fluidized beds are extensively used to remove the byproduct HCl from the fumed oxides (deacidification), or for chemical modification of the surface groups, for example, to make hydrophilic fumed silica hydrophobic (Flesch et al. 2008).

Oxygen-containing groups on the surface of carbon black particles strongly influence their properties, such as vulcanization rate, flow characteristics, and color. Oxidative after treatment of carbon black in a fluidized 1633 bed system can be used to tune these properties (Voll and Kleinschmit 2000). However, it is anticipated that in the near future, NPs will be applied much more broadly. It will be crucial to scale-up production processes while precisely maintaining the specifications of the particulate product. We expect that fluidization can play an important role in both the production and application of NPs, as it can be used for operation such as reaction, coating, granulation, mixing, drying, and adsorption.

Currently, NPs are already applied in, for example, chemical–mechanical polishing, in powder flow enhancement, in catalysis, and in medicine. In most of these applications, fluidization does not play (yet) a large role. NPs are used for chemical–mechanical polishing in the fabrication of semiconductor chips to prevent microscratching (Singh et al. 2002; Yang 2005). NPs are also used as a flowing aid for larger particles: coating cohesive micron-sized particles with NPs can significantly increase the flowability of cohesive powders (Yang et al. 2005; Linsenbühler and Wirth 2002; van Ommen et al. 2010b; Valverde et al. 1998).

Most heterogeneous catalysts consist of nanosized particles dispersed on a high surface area support. However, most catalysts of industrial importance have been developed by trial-and-error experimentation (Jacobsen et al. 2001). A better scientific basis could make catalyst development substantially more efficient. For example, advances in characterization methods have led to a better understanding of the relationships between NP properties and catalytic performance (Bell 2003).

NPs play an increasing role in medicine, both for imaging or for transporting and delivering therapeutic agents (Jain 2007; Medina et al. 2007). Coating of nanosized drug particles with certain biodegradable polymers will allow controlled release, protect it from stomach acids, and prevent it from becoming trapped in a mucus barrier so it can be targeted to specific organs of the body (Lai et al. 2008), and prevent immune cells (macrophages) from engulfing and eliminating the nanosized drug particles circulating in the bloodstream. The application of NPs also offers new possibilities toward the development of personalized medicine (Riehemann et al. 2009).

A potential use of NPs is in enhanced calcium-based sorbents for CO_2_-capture (Li et al. 2010; Lu et al. 2009). Alternatively, silica nanopowder can be mixed with calcium hydroxide fine powder to enhance the efficiency of CO_2_ adsorption by improving the gas–solids contact efficiency in a fluidized bed (Valverde et al. 2011). In this case, uniformly fluidizable agglomerates of silica NPs serve as carriers of Geldart C particles with high CO_2_ adsorption capacity.

In several applications, core−shell NPs exhibit superior physical and chemical properties compared to their single-component counterparts (Zhong and Maye 2001; Caruso 2001); fluidization can play an important role in making such particles. The combination of two or more materials gives additional degrees of freedom in the creation of NPs and consequently an enormous amount of potential particle structures. Up to now, most attention in literature is aimed at liquid-phase methods for synthesizing core−shell NPs. These methods typically yield only small amounts of material: they are cumbersome to scale up. Moreover, such recipes are often very specific for just one type of core−shell NP. Gas phase methods can more easily produce larger amounts of material and are typically more generic (Strobel and Pratsinis 2007; Ullmann et al. 2002). A successful technique to make nanostructured particles of various compositions in the gas phase is flame spray pyrolysis (Dosev et al. 2007; Kim and Laine 2009; Teleki et al. 2008a). An advantage of this method is that NP production and coating are carried out in a single step; a disadvantage is that rather wide particle size distributions are obtained. An alternative is to separate the synthesis of core and shell into two subsequent steps. There are several techniques available to coat NPs in a fluidized bed process. These techniques will be discussed below.

A common technique for gas-phase coating objects with a closed layer is chemical vapor deposition (CVD). In a typical CVD process, the substrate is exposed to one or more gaseous precursors, which react on a surface to produce the desired film. CVD is commonly used in the semiconductor industry, but can also be used to produce coated particles, e.g., noble metal catalyst particles and layered luminescent pigments (Czok and Werther 2006). However, CVD is less suited to coat NPs. Since different chemical reactants coexist in the gas phase during the CVD reaction, homogeneous reactions can take place that form NPs contaminating the product. Moreover, truly uniform and conformal films on individual NPs have not been achieved (Hakim et al. 2005a).

Instead of CVD, ALD can provide particles with an ultra-thin, uniform layer. This technique is different from CVD in that the chemistry is split into two half-reactions: the different reactant gases are fed to the sample consecutively rather than simultaneously. For example, for an alumina coating process, a precursor such as tri-methyl-aluminum binding to the surface by chemisorption in step (A) reacts with an oxidizer such as water in step (B). A simplified version of the reaction scheme is (Puurunen 2005):

15 $$ \begin{gathered} ({\text{A}}) \, \left\|
{{\text{Al}}{-}{\text{OH}}} \right. \, + {\text{
Al}}({\text{CH}}_{3} )_{3} \, ({\text{g}}) \, \longrightarrow \,
\left\| {{\text{Al}}{-}{\text{O}}{-}{\text{Al}}({\text{CH}}_{3}
)_{2 \, } } \right. + {\text{ CH}}_{4} \, ({\text{g}}) \hfill \\
({\text{B}}) \, \left\| {{\text{Al}}{-}{\text{CH}}_{3} } \right.
\, + {\text{ H}}_{2} {\text{O }}({\text{g}}) \, \longrightarrow \,
\left\| {{\text{Al}}{-}{\text{OH}}} \right. \, + {\text{ CH}}_{4}
\, ({\text{g}}) \hfill \\ \end{gathered} $$

where ║ denotes the solid surface. The number of times the (A)–(B) cycle is repeated determines the thickness of the coating, resulting in full control over the layer thickness at the atomic level.

**Fig. 13:**
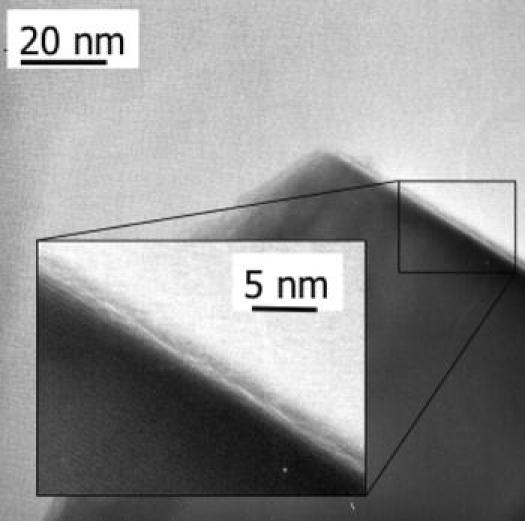
TEM picture of a LiMn_2_O_4_ particle coated with a thin layer of alumina (five ALD cycles) at atmospheric pressure. Such NPs can be used as cathode material in Li-ion batteries (reprinted from van Ommen et al. (2010b) with permission from Elsevier)

ALD can be applied to a wide range of particles sizes (~10 nm–500 μm) and materials. Weimer and co-workers (Ferguson et al. 2000; Hakim et al. 2005a) showed that applying ALD to particles is best carried out when these particles are fluidized. In the semi-conductor industry, ALD is typically carried out under vacuum to enhance the removal of non-reacted precursors and gaseous by-products. Typically Weimer and co-workers apply ALD to particles at low pressure, ~100 Pa. However, Beetstra et al. 2009 showed that ALD of fluidized particles can also be carried out at atmospheric pressure (see Fig. 13), which simplifies the fluidization of the particles and facilitates process scale up.

Molecular layer deposition is a technique related to ALD; with this coating technique organic layers instead of inorganic layers are deposited (Liang et al. 2009). Several authors have been using plasma-enhanced CVD to provide micron sized and NPs with a very thin layer (Jung et al. 2004; Sanchez et al. 2001; Spillmann et al. 2006; Abadjieva et al. 2011), although only Spillmann et al. (2006) coated NPs. Esmaeili et al. (2009) used a fluidized bed reactor for encapsulating NPs by few nm of polyethylene using Ziegler−Natta catalysts.

We anticipate that in the coming years, NPs will find more and more applications in medicine, catalysis, and energy processes. In some cases simple, single-material NPs can be applied, but several applications ask for more complex, nanostructured particles such as core−shell particles. It will be crucial to scale-up production processes while precisely maintaining the specifications of the particulate product. We believe that fluidization of NPs will play an important role in this. A strong interplay between different disciplines, including physical chemistry, material sciences, reaction engineering, and fluid mechanics, is essential for reaching important breakthroughs in the manufacturing and processing of NPs.

Proper fluidization of NPs is often not possible without an assistance method. As discussed earlier, we think that the use of microjets is the most promising approach. However, the exact working mechanism of these microjets is not yet fully understood. Also some of the other assistance methods, such as the use of acoustic waves, need further research to fully understand and optimize them. Another virtually unexplored field is the modeling of reactions involving fluidized nanopowders. Given the large range of length scales that play a role—one NP agglomerate easily consists of billions of particles—a multi-scale modeling approach will be needed.

The increased use of NPs will also require more attention for the safe and sustainable use of these materials. Although humans have been exposed to airborne NPs throughout their evolutionary stages, such exposure has increased dramatically over the last century due to anthropogenic sources such as combustion processes. The increasing use of engineered nanomaterials is likely to become yet another source through inhalation, ingestion, skin uptake, and injection of engineered nanomaterials, requiring more information about safety and potential hazards of NPs (Oberdörster et al. 2005). According to Nel et al. (2006), a proactive approach is required in safety evaluations, and the regulatory decisions should follow from there. In addition to facilitating the safe manufacture and implementation of engineered nanoproducts, these authors foresee also potential positive spin-offs of the understanding of nanotoxicity. For instance, the propensity of some NPs to target mitochondria and initiate programmed cell death could be used as a new cancer chemotherapy principle. Auffan et al. (2009) conclude on basis of a literature study that “larger” NPs (30–100 nm) show merely the same behavior as bulk materials, while NPs smaller than 30 nm have unique properties that require specific regulations.

## Conclusions

Fluidization can be used to process large quantities of nanopowders in the gas phase. The NPs are not fluidized as individual particles, but as agglomerates. Because of interparticle forces such as van der Waals forces and capillary forces, agglomerates are formed, which are very dilute and have a fractal nature. The agglomerates are typically a few hundred μm in size and have a voidage of about 0.9–0.99. Regular fluidization of these nanopowders can lead to two different types of fluidization: APF (agglomerate particulate fluidization) and ABF (agglomerate bubbling fluidization). APF is smooth, liquid-like, bubble-less fluidization that is only observed for certain types of NPs and aerogels. ABF is bubbling fluidization with very little bed expansion, as also observed for other small particles of Geldart type C.

To enhance the fluidization of nanopowders—especially those of the ABF type—various assistance techniques can be used: mechanical vibration, mechanical stirring, sound waves, pulsed gas flow, a centrifugal field (rotating fluidized bed), alternating electric field, or secondary gas injection using microjets. The techniques typically lead to mixing at the micron-scale: parts of agglomerates are exchanged. Only the use of microjets has been shown to lead to mixing of individual NPs, but more research needs to be done to verify this observation.

Several approaches have been applied to model the behavior of fluidized nanopowders. A force balance can be used to calculate the average size of NP agglomerates in a fluidized bed, also when additional external forces (e.g., due to vibration) are exerted. An alternative is to use a modified Richardson and Zaki equation to estimate the agglomerate size. Some first attempts have been made to apply CFD, either using an Eulerian−Eulerian approach requiring specific closures to describe the agglomerates as a continuous phase, or by discrete element modeling in which the individual agglomerates are modeled.

The application of nanopowder fluidization in practice is still limited, but a wide range of potential applications is foreseen, e.g., in medicine, catalysis, and energy processes. For many applications, advanced materials incorporating NPs will needed, and fluidization is a convenient way to transport and mix them, or process them in some other way. Fluidized beds can also be applied to provide NPs with a thin coating, obtaining core−shell NPs. Using fluidization, it is possible to process large amount of NPs, which is convenient for applications which will require NPs on the ton-scale, such as catalysis and energy conversion and storage. We expect that both the unsolved scientific challenges and technological questions arising from novel applications will boost research in nanopowder fluidization in the coming years.

## Electronic supplementary material

Below is the link to the electronic supplementary material.

Supplementary material 1 (WMV 1,927 kb)

Supplementary material 2 (WMV 6,151 kb)
